# The Treatment of Strangulated Hernia

**Published:** 1893-02-04

**Authors:** 


### ST. BARTHOLOMEW'S HOSPITAL.

### The Treatment of Strangulated Hernia.

Strangulated hernia being certainly one of the most common, if not actually the most common, of the surgical emergencies which a practitioner has to face, its treatment is, therefore, of the utmost importance.

At St. Bartholomew's Hospital the cases are, as a rule, first brought to the surgery, and there seen by the house surgeon on duty. They may be roughly divided into two classes, viz., those that have already been under treatment, and those that have not. This division is mentioned here, because very often it has a direct bearing on the subsequent treatment and course of the case; for it is still unfortunately the fact, that every year a certain number of patients with strangulated hernia have before admission been treated either with purgative medicine or by violent attempts at reduction by taxis, in which case no further taxis is applied, but the patient at once submitted to operation. In the surgery usually an attempt is made, if the patient be in good condition, to reduce the hernia by taxis, and this is not infrequently successful, in which case a pad of lint and a spica bandage are applied, and the patient advised to get a truss, either at the Truss Society or elsewhere, as soon as he possibly can.

If taxis fails in the surgery, the patient is then admitted into the wards, where a subsequent attempt may be made, with, perhaps, the additional help of placing the patient in a hot bath for ten minutes or so, and then attempting reduction while still in the bath. In applying taxis the following points must be thought of:—

1. Has the patient been previously submitted to this form of treatment, and, if so, for how long? as prolonged taxis endangers the gut, and may cause its rupture or gangrene, especially in femoral hernia. If a previous attempt has been made, as a rule very little further effort is expended, and, at any rate, only for a very short time.

2. The patient must be placed in such a position as to relax the openings through which the hernia comes down, by putting him on his back with the legs raised and adducted so as to relax the abdominal walls and the parts about the ring. The neck of the sac must then be steadied by the left hand, and at the same time somewhat drawn down, while steady pressure is made on the sac and its contents. The first result will probably be to displace some of the fluid in the sac, and then the contents may slip steadily into the abdomen with a gurgle. On no account must the sac, &c., be pushed up forcibly towards the abdomen, as this cannot produce the desired result, and only tends to reduce the sac as well as the gut through the ring, producing "reduction *en masse*," and leaving the strangulation at the neck of the sac unrelieved.

If taxis as above described fails, one of the senior surgeons is then sent for to see the case, and almost always the patient is removed to the operating theatre immediately on his arrival, and an anæsthetic administered, when generally a short attempt is again made to reduce by taxis, and if this be unsuccessful operative treatment is at once proceeded with.

The parts are shaved and cleansed with soap and water, and subsequently with an antiseptic lotion (usually Lotio Hydrargyri Perchloridi, 1-2,000). An incision is made from two to three inches in length over the neck of the sac, and the sac opened; as this is nearly always tense with fluid, there is but little danger of wounding the gut, though such an accident may happen. If it does, the opening is at once stitched with fine silk by Lambert's suture, and ill results very rarely follow. The gut is now drawn on one side by the left hand, while one finger is passed up to the stricture, and along this finger the hernia knife is passed, and the stricture divided. The loop of gut is then gently drawn down so that the part strictured can be examined, and if sufficiently sound the gut is returned into the abdomen.

A word must here be said about the way the omentum is treated. It is usually not returned, but transfixed with an aneurism needle armed with carbolised silk; the loop is then divided, and the piece of omentum tied in two parts, and that below the ligature removed, the stump being returned to the abdomen. Care must be taken not to treat the omentum roughly, lest some of its large veins in the abdomen be ruptured, causing dangerous bleeding. After the contents have been reduced it is now the practice at St. Bartholomew's to complete the operation by doing a radical cure, except where the constitutional condition of the patient is so grave as to require a rapid completion of the operation.

The steps of the radical cure vary so much in detail that they cannot be described here; but in the main they all consist in either removing the sac after transfixing its neck and tying in two parts, or drawing the sac up into a folded mass, which passes up into the internal ring, and then subsequently closing the vaginal canal by stitching the conjoint tendon to Poupart's ligament and closing the external ring in the same way. In femoral hernia the femoral ring is closed by sutures after the sac has been treated as above. The sutures most commonly used are silver wire and carbolised silk, the former being most in favour.

The treatment of the gut which is unfit to be returned into the abdomen, must now be considered; and, first, a few words must be said as to the test of fitness. When a knuckle of intestine, though much congested and purple in colour, retains, to some extent, the polish of its peritoneal coat, it may be considered fit to return: but in this condition it must be left as near to the internal ring as possible. If, however, the gut be actually black and gangrenous there are three courses open, viz.:—

1. To lay open the gut and leave the structure undivided, so as not to disturb any peritoneal adhesions that may have been formed.

2. To divide the stricture, and then lay the gut freely open.

3. To do a primary resection of the damaged piece of gut.

The second plan is the one usually adopted at St. Bartholomew's while the third is, we believe, advocated by some of the staff, in suitable cases, although the cases of gangrene are usually in much too collapsed a condition to allow of a prolonged operation. By either the first or second method an artificial anus results, which must be subsequently treated.

The after-treatment of an ordinary uncomplicated case of strangulated hernia is generally carried out at St. Bartholomew's as follows:—

Immediately after the operation the patient is returned to bed, and placed on the back with the legs flexed over a pillow, and, as far as is possible, no food is given for the first twenty-four hours, thirst being allayed either by teaspoonfuls of *hot* water or by small pieces of ice. At the end of this time food is commenced in the form of small quantities of milk and water. It may, however, be necessary to give food before this if the patient be very collapsed, and in that case a little brandy is given as well. As to drugs, tinct. opii. or liq. opii. sedativus are the two which are most used, but neither are necessary unless the patient be either in pain or restless and sleepless. It must also not be forgotten that it is often necessary to draw off the water for the first day or so. As to the bowels, they are generally left to act by themselves; but if they are not moved before about the eighth day a small soap and water enema is generally given to assist them. In some cases diarrhœa supervenes rapidly after the operation. This is usually the case in patients who have been previously treated with purgatives. In these cases opium and bismuth are the two drugs which are most given, and they must be given persistently. Stimulants will also be necessary, as these patients get very collapsed and not infrequently die.

Previous to the departure of a patient from the hospital, a truss is fitted, and this is done by almost all the surgeons, even when a radical cure was carried out at the time of the operation. The patients are also warned as to the risk they run if a hernia is allowed to be frequently down and is not efficiently kept up by a truss.

In closing this article, it need hardly be said that it has been impossible within its narrow limits to treat such a large subject exhaustively, but enough has been said to give an outline of the treatment of strangulated hernia at St. Bartholomew's.

